# Phage Biocontrol of *Campylobacter jejuni* in Chickens Does Not Produce Collateral Effects on the Gut Microbiota

**DOI:** 10.3389/fmicb.2019.00476

**Published:** 2019-03-12

**Authors:** Philip J. Richards, Phillippa L. Connerton, Ian F. Connerton

**Affiliations:** Division of Food Sciences, School of Biosciences, University of Nottingham, Loughborough, United Kingdom

**Keywords:** bacteriophage, campylobacter, microbiota, chicken, biocontrol

## Abstract

Bacteriophage biocontrol to reduce *Campylobacter jejuni* levels in chickens can reduce human exposure and disease acquired through the consumption of contaminated poultry products. Investigating changes in the chicken microbiota during phage treatment has not previously been undertaken but is crucial to understanding the system-wide effects of such treatments to establish a sustainable application. A phage cocktail containing two virulent *Campylobacter* phages was used to treat broiler chickens colonized with *C. jejuni* HPC5. *Campylobacter* counts from cecal contents were significantly reduced throughout the experimental period but were most effective 2 days post-treatment showing a reduction of 2.4 log_10_ CFU g^-1^ relative to mock-treated *Campylobacter* colonized controls. The administered phages replicated *in vivo* to establish stable populations. Bacteriophage predation of *C. jejuni* was not found to affect the microbiota structure but selectively reduced the relative abundance of *C. jejuni* without affecting other bacteria.

## Introduction

*Campylobacter* ssp. cause foodborne illness worldwide (Kaakoush et al., 2015) and represent the most commonly reported zoonotic pathogens in the European Union with 246,307 confirmed cases of gastrointestinal illness in 2016 (European Food Safety Authority [EFSA], 2017). *Campylobacter jejuni* is the most common species causing human disease (83.6%), but the burden of disease caused by *Campylobacter coli* is also significant (8.5%) (European Food Safety Authority [EFSA], 2017). Both species readily colonize the poultry gut, where the impact on flock health and production parameters varies with husbandry practice and the colonizing organism (Gormley et al., 2014; Humphrey et al., 2015). Estimates of broiler chicken flock prevalence vary between nations with 0.6 to 13.1% in the Nordic countries up to 74.2–80% in other European countries (European Food Safety Authority [EFSA], 2010a). Source attribution studies have predicted that up to 80% of human illness is as a result of exposure to campylobacter arising from poultry sources (European Food Safety Authority [EFSA], 2010b). A recent source attribution study referenced at the point of exposure confirmed chicken meat as the most important source of *Campylobacter* enteric disease, with an estimated 65–69% of human campylobacteriosis cases (Ravel et al., 2017). Poultry meat contaminated with digesta during slaughter/processing therefore represents a significant risk to public health (Osimani et al., 2017).

Strict on-farm biosecurity measures to mitigate the *Campylobacter* colonization of poultry have been implemented in many countries, but these alone do not eliminate campylobacter from poultry. Additional to these efforts, intervention strategies have been developed to reduce the *Campylobacter* colonization levels of poultry, which have the potential to reduce human exposure if the reductions can be realized on poultry meat (Rosenquist et al., 2003; Newell et al., 2011). The use of *Campylobacter*-specific bacteriophages (commonly referred to as phages) to reduce the intestinal carriage of *Campylobacter* by broiler chickens is one such intervention that has shown promise in controlled trials (Loc Carrillo et al., 2005; Wagenaar et al., 2005; El-Shibiny et al., 2009) and in broiler house applications (Kittler et al., 2013). These studies have been conducted with phage applications of one or more phage to achieve reductions in the cecal counts of *Campylobacter* colonized chickens of approximately 2 log_10_ CFU g^-1^ (Carvalho et al., 2010; Connerton et al., 2011; Hammerl et al., 2014).

The use of multiple types of phage targeting different receptors in the form of phage cocktails offers the prospect of expanding the host range of the preparation whilst limiting the potential to develop resistance to all components of the cocktail (Chan et al., 2013). This has been explored experimentally using *Campylobacter*-specific phage cocktails containing either four (Fischer et al., 2013) or two phages (Hammerl et al., 2014). In both cases phages were selected from both Group II and Group III as classified by Sails et al. (1998). The classification was originally based on total genome size and morphology but it is now known that the two groups share little DNA similarity and generally have different host specificities (Javed et al., 2014; Jäckel et al., 2017). Group II and III *Campylobacter* bacteriophages are taxonomically classified as members of the *Myoviridae* subfamily *Eucampyvirinae*, which do not possess the genes required to form stable lysogens and therefore exhibit a lytic or virulent life cycle similar to most Myoviruses (Javed et al., 2014). *Campylobacter* phage isolated using *C. jejuni* and *C. coli* hosts may show some cross-species specificity within strains of *C. jejuni* and *C. coli* species but have not been demonstrated to infect other species in the *Campylobacter* genus, for example *C. lari*, *C. upsaliensis*, *C. fetus*, *C. Sputorum*, or *C. intestinalis* (Loc Carrillo et al., 2007). Neither have they been found to infect *Helicobacter pylori*, *Arcobacter butzleri*, *Citrobacter freundii*, *Salmonella enteritica* serovar Enteritidis, *Escherichia coli*, and *Pseudomonas aeruginosa* (Loc Carrillo et al., 2007). Therefore it may be anticipated that components of a *Campylobacter* phage cocktail would retain their specificity and not give rise to dysbiosis – a detrimental microbiota constitution that often arises post broad-spectrum antibiotic therapies (Dethlefsen et al., 2008). However, a recent study has suggested that exposure of the rat gut microbiome to a cocktail of commercial phage preparations active against *Salmonella enterica*, *Staphylococcus aureus*, *Streptococcus pyogenes*, *Proteus mirabilis*, *P. vulgaris*, *Pseudomonas aeruginosa*, *Klebsiella pneumoniae*, and *E. coli* results in dysbiosis with increased inflammation and gut permeability (Tetz et al., 2017). Bacteriophage mediated cell lysis has the potential to release lipopolysaccharides (endotoxin) from Gram-negative bacterial species that are potent inducers of proinflammatory cytokines in animals and in humans (Medzhitov, 2007). Exposure to a phage cocktail targeting multiple Gram-negative species may well elicit such a response, although it is reported that targeted virulent *E. coli* bacteriophages release less endotoxin than β-lactam antibiotics (Dufour et al., 2017), and that the phage tail adhesin protein Gp12 can bind free lipopolysaccharide to counteract the inflammatory effect (Miernikiewicz et al., 2016). Typically the lipopolysaccharides of Gram-negative bacteria are composed of an outer membrane anchored lipid A substituted with a core polysaccharide structure attached to a set of repeating O-chain subunits. However, *C. jejuni* synthesizes a core oligosaccharide without the repeating O-chain, which is referred to as lipooligosaccharide (Karlyshev et al., 2005).

In this study we demonstrate that *Campylobacter* phage affect the target host bacteria without provoking intestinal dysbiosis, when phages are administered to broiler chickens at therapeutic doses.

## Methodology

### Bacterial Strains and Growth Media

Campylobacter were isolated and enumerated by direct plating on modified Cefoperazone Charcoal Deoxycholate Agar (mCCDA) selective medium (Oxoid, Basingstoke, United Kingdom) with addition of cefoperazone and amphotericin B selective supplement (Oxoid) using standard techniques. *C. jejuni* HPC5 was isolated from the cecal content of a commercial broiler chicken in the United Kingdom (Loc Carrillo et al., 2005; NCBI accession CP032316). The universal bacteriophage host strain *C. jejuni* PT14 was used to propagate *Campylobacter* bacteriophages (Brathwaite et al., 2013; NCBI accession CP003871). *C. coli* NCTC 12668 was used to discriminate group II and III bacteriophages (Frost et al., 1999). *C. jejuni* HPC5 for the inoculation of broiler chickens and campylobacter for the production of bacterial lawns were cultured on horse blood agar (Blood agar base No 2; Oxoid) with addition of plus 5% (v/v) defibrinated horse blood, (TCS, Buckingham, United Kingdom) under microaerobic conditions (5% O_2_, 5% H_2_, 10% CO_2_, 80% N_2_, produced by the evacuation and replacement technique) at 42°C for 24 h.

### Bacteriophages and Propagation

*Campylobacter*-specific bacteriophages CP20 and CP30A were isolated from commercial broiler chicken excreta collected in the United Kingdom in 2001. Bacteriophages were isolated by making a 10% suspension of excreta in SM buffer (50 mM Tris–HCl, pH 7.5, 0.1 M NaCl, 8 mM MgSO_4_.7H_2_O, and 0.01% gelatin; Sigma Aldrich, Gillingham, United Kingdom). This was incubated at 4°C with agitation, for 24 h followed by centrifugation at 13,000 × *g* for 5 min to remove bacteria. The resulting supernatant was filtered through a 0.2 μm filter (Minisart, Sartorius, Goettingen, Germany) to remove any remaining bacteria.

When screening for the presence or absence of bacteriophage, 10 μl filtrate aliquots were applied to bacterial lawns of *C. jejuni* PT14 prepared using the soft agar overlay method as previously described (Connerton et al., 2004). Bacteriophage were propagated by complete plate lysis and recovered in SM buffer before filtration (0.2 μm filter) and concentration by centrifugation at 37,000 × *g*.

CP20 (NCBI nucleotide accession MK408758) corresponds to a group II *Campylobacter* bacteriophage and CP30A (NCBI nucleotide accession JX569801) a group III based on genome sizes, capsid morphologies determined by transmission electron microscopy and genomic DNA sequences (Scott et al., 2007a; Siringan et al., 2011, 2014; Javed et al., 2014; Brathwaite et al., 2015).

### Experimental Birds

Commercial *Campylobacter*-free male Ross 308 broiler chicks were obtained as hatchlings (PD Hook, Oxfordshire, United Kingdom). Birds were housed in a controlled environment in individual pens under strict conditions of biosecurity. Temperatures were as outlined in the Code of Practice for the Housing and Care of Animals Bred, Supplied or Used for Scientific Purposes. Birds were provided with commercial broiler diets (starter, grower, and finisher) and water *ad libitum* for the duration. The birds were weighed and randomly assigned to 3 groups at 14 days of age. These were: Control group consisting of *Campylobacter*-free sentinel birds, Group Cj_phg, consisting of *Campylobacter* infected birds to be administered with phage and Group Cj, consisting of *Campylobacter* infected birds to be administered with placebo instead of phage (mock treatment). Cloacal swabs were taken on day 14 and tested for *Salmonella* by direct plating on Xylose-Lysine desoxycholoate agar (XLD) agar (Oxoid) and for *Campylobacter* by direct plating on mCCDA agar. Excreta samples, from the same day, were tested for *Campylobacter* phage (see below) and for *Salmonella* by enrichment in Rappaport-Vassiliadis soya peptone broth (Oxoid) then plating on Xylose-Lysine desoxycholoate agar (XLD) agar (Oxoid). *C. jejuni* HPC5 has previously been demonstrated to establish intestinal colonization of Ross 308 broiler chickens at 20 days of age within 48 h of oral gavage, and to maintain colonization levels without significant differences in cecal counts over 15 days (Loc Carrillo et al., 2005; Connerton et al., 2018). Four days post oral gavage was selected for phage administration to ensure intestinal colonization, and that any variation in the cecal counts would be evident over the period of the experiment. Birds from Cj and Cj_phg groups were colonized with *C. jejuni* HPC5 at 20 days of age. Each bird received 7 log_10_ CFU *C. jejuni* in 1 ml of PBS (phosphate buffered saline) by oral gavage. Phages were administered to Group Cj_phg as a single dose of log_10_ 7 PFU of CP20 and CP30A combined in 1 ml of 30% CaCO_3_ (antacid) by oral gavage at 24 days of age. Group Cj were administered with 1 ml of 30% CaCO_3_ as a placebo at 24 days of age. Five birds were sacrificed at 24 h intervals from 25 to 29 days of age following administration of phage or placebo. The ceca, ileum, and colon of the birds were first separated by ligature and then removed by sterile dissection. The luminal contents were collected for *Campylobacter* and bacteriophage isolation as described below and aliquots stored at -80°C for DNA extraction.

### Enumeration of Campylobacters

Serial dilutions of digesta were made in maximum recovery diluent (MRD; Oxoid) and enumerated using the Miles and Misera technique on mCCDA agar with 2% (w/v) additional agar to reduce swarming. Plates were incubated under microaerobic conditions at 42°C for 48 h before typical *Campylobacter* colonies were counted.

### Enumeration of Bacteriophages

Bacteriophages were recovered by making a 10% suspension of chicken digesta in SM buffer as described above. To enumerate bacteriophage CP20 and CP30A in the phage treated groups, independent lawns of *C. jejuni* HPC5 and *C. coli* NCTC 12668 were prepared. The *C. coli* strain NCTC 12668 was used as a second host because it was sensitive to CP20 but not CP30A, which allowed discrimination of the two phages administered. The CP30A titers were obtained by subtraction of the CP20 titer on *C. coli* 12668, from the total phage count on the *C. jejuni* HPC5 host, which was sensitive to both phages. Serial dilutions of intestinal contents were prepared in SM buffer and applied to these lawns as 10 μl spots in triplicate. The plates were then incubated for 24 h at 42°C under microaerobic conditions.

### Acquisition of Bacteriophage Resistance

In order to establish the frequency of resistance to bacteriophages post-intervention, single colonies were lifted from the primary isolation plates onto which cecal content from all phage-treated and mock-treated control birds had been inoculated. Three colonies per cecal sample were picked for each phage-treated bird and subcultured on horse blood agar plates. Bacterial lawns were prepared from successful subcultures and CP20 and CP30A phage were applied at a range of dilutions from 1 to 3 log_10_ PFU to establish if resistance had been acquired with respect to the efficiency of plating.

### DNA Isolation

DNA was isolated from both ileal and cecal content using the Mobio PowerSoil kit (now QIAGEN Ltd., Manchester, United Kingdom). The method used is as described in the Human Microbiome Project SOP for processing of stool Specimens (see Manual of Procedures for Human Microbiome v12, section 7.7 onward^1^.

### Microbiome Analysis

The V4 regions of the bacterial 16S rRNA genes were PCR amplified using the primers 515f (5′ GTGCCAGCMGCCGCGGTAA 3′) and 806r (5′ GGACTACHVGGGTWTCTAAT 3′) (Caporaso et al., 2011). Amplicons were then sequenced on the Illumina MiSeq platform using 2 × 250 bp cycles. The 16S rRNA gene sequences were quality filtered and clustered into operational taxonomic units (OTUs) in Mothur (Schloss et al., 2009) using the Schloss lab. MiSeq SOP^2^ (Kozich et al., 2013). Batch files of Mothur commands used in this study are available at: https://github.com/PJRichards. Post-processing rarefaction curves were plotted to assess sampling effort (Supplementary Figures S1–S3). Based on these observations the Day 1 (1dpt) ileum communities and two further ileum communities (Group Cj: Day 2 replicate 2; Group Cj_phg: day 4 replicate 4) were judged as having insufficient depth, and were therefore excluded from the analysis. No template controls were included in the analysis.

Raw sequence data are deposited in the NCBI database within the Bioproject PRJNA506577 under the SRA study SRP170194.

### Ethics Statement

All experimental animal work was performed in accordance with United Kingdom and EU law. This study was approved by the Local Ethics Committee of the University of Nottingham and performed under Home Office license.

### Statistical Analysis

The article was written in R 3.5.1 (R Core Team, 2018) and Rmarkdown 1.10.8 (Allaire et al., 2018; Xie et al., 2018) using Rstudio 1.1.456 (RStudio Team, 2015). R code used to make the figures/tables presented here are available at: https://github.com/PJRichards/Richards_phage_microbiota. Figure 3 was drawn in R using code adapted from Torondel et al. (2016) and kindly made available at: http://userweb.eng.gla.ac.uk/umer.ijaz. OTUs discriminatory between communities were identified using LEfSE (Segata et al., 2011). Commonality of 16S rDNA sequences of OTUs discriminative of phage-treated (Group Cj_phg) and untreated birds (Group Cj) with other trials performed in our laboratory were determined using stand-alone ncbi-blast-2.7.1+ (Zhang et al., 2000)^3^.

## Results

### Dual *Campylobacter* Bacteriophage Treatment of Reduces Levels of *Campylobacter jejuni* Colonization

*Campylobacter* were enumerated using standard culture methods from intestinal luminal contents collected from the ileum, ceca, and colon of all birds. High levels of *Campylobacter* were recovered from all digesta collected from *C. jejuni* colonized birds (Group Cj) throughout the 5-day period of the experiment (mean log_10_ CFU g^-1^: ileum 6.289, ceca 8.000, colon 7.452 for *n* = 25). A control group comprising of a cohort of non-colonized sentinel birds confirmed the effectiveness of the biosecurity measures adopted as they remained free of *Campylobacter* and phage contamination.

**FIGURE 1:**
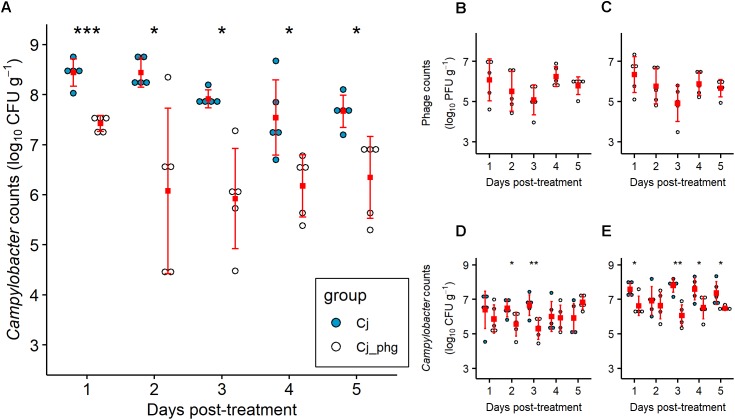
*Campylobacter jejuni* levels are reduced by phage predation post-treatment. Independently housed Ross 308 broilers were given 7 log_10_ CFU *C. jejuni* by oral gavage at 20 days-old. At 24 days-old birds were orally administered a mixture of two phage at 7 log_10_ PFU each or a placebo of carrier alone. Five chickens from each group were sacrificed on each day from 25 to 29 days-old, from which *C. jejuni* were enumerated from the intestinal contents of the ceca **(A)**, ileum **(D)**, and colon **(E)**. Titers of phage CP20 **(B)** and CP30A **(C)** were determined from cecal content. Filled squares indicate means. Error bars indicate standard deviation. Asterix indicate statistical significance: ^∗^, *p* ≤ 0.05; ^∗∗^*p* ≤ 0.01; ^∗∗∗^*p* ≤ 0.001.

Co-administration of a single phage dose containing CP20 (7 log_10_ PFU) and CP30A (7 log_10_ PFU) 4 days after *Campylobacter* exposure at 24 days of age, significantly reduced *C. jejuni* numbers in the ceca of phage-treated birds (Group Cj_phg) compared to mock-treated birds throughout the period of the experiment (*p* ≤ 0.032; Figure 1A). The phages were most effective 2 days post-treatment (dpt), resulting in a reduction in *C. jejuni* numbers of 2.365 log_10_ CFU g^-1^ in Group Cj_phg compared to *C. jejuni* colonized controls in Group Cj. After this time, the numbers of *C. jejuni* in Group Cj_phg birds increased but remained significantly lower than the levels observed from mock-treated birds (Group Cj) by 1.321 log_10_ CFU g^-1^ after 5 days.

Phage treatment was most effective in the cecum, the organ that represents the greatest biomass of digesta and reservoir of *C. jejuni*. However, treatment was also effective in reducing *C. jejuni* in the ileum (*p* = 0.044 and 0.008 at 2 and 3 dpt, respectively) and the colon at all days excluding 2 dpt (*p* ≤ 0.041; Figure 1E). The reductions in *Campylobacter* counts were not as great as those observed for the ceca, with a maximum reduction in the ileum of 1.359 log_10_ CFU g^-1^ at 3 dpt and a maximum reduction in the colon of 1.740 log_10_ CFU g^-1^ also at 3 dpt (Figure 1D,E). Bacteriophages were enumerated over the 5 day trial period and were detected in the cecal contents of all 5 birds treated in Group Cj_phg from 24 h after administration (Figure 1B,C). The CP20 phage titre recovered from cecal contents of the treated birds remained stable over time (mean 5.738 log_10_ PFU g^-1^; *SD* 0.460; Figure 1B), confirming that the phage were replicating *in vivo*. Similarly, the mean CP30A titre was 5.708 log_10_ PFU g^-1^ (*SD* 0.517; Figure 1C) confirming the two phages co-exist without competitive exclusion. The levels of phages recovered from ileum and colon contents also remained stable over the course of the experiment (Supplementary Figure S4).

### Bacteriophage Resistance Post Treatment

The overall levels of phage resistance (isolates resistant to one or both phage) in *C. jejuni* HPC5 isolates in Group Cj_phg was approximately 10% (*n* = 7/67) of the strains recovered post-treatment. Of these, three (4.5%) were resistant to both CP20 and CP30A phages whilst one (1.5 %) was resistant to CP20 but not CP30A and three (4.5%) were resistant to CP30A only. No phage resistance was detected in the *C. jejuni* recovered from birds that had not received phage (*n* = 32).

### Bacteriophage Predation of *Campylobacter jejuni* Does Not Affect Microbiota Structure

**FIGURE 2:**
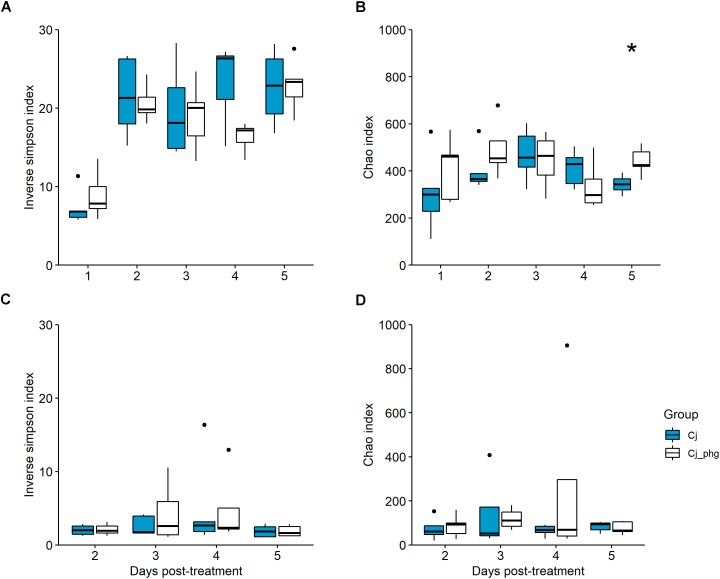
Bacterial diversity within the gastrointestinal tract is not affected by phage treatment. Box-and-whisker plot describing bacterial 16S rDNA gene content surveys of cecal lumen contents from phage-treated (Cj_phg) or mock-untreated (Cj) groups in terms of α-diversity (inverse Simpson’s index; **(A)** and richness (Chao index; **(B)**. Corresponding α-diversity and richness for ileal lumen contents are shown in panels **(C,D)**, respectively. Asterix indicate statistical significance: ^∗^*p* ≤ 0.05.

The α-diversity (inverse Simpson index) of the cecal or ileal microbiota of bacteriophage-treated birds (Group Cj_phg) were not significantly different to those from mock-treated birds (Group Cj) (*p* ≥ 0.095 and ≥0.841, respectively; Figure 2A,C). There was no difference in the richness (Chao) of the cecal microbiota between 1 and 4 dpt (*p* ≥ 0.151) or the ileum microbiota at any time (*p* ≥ 0.548; Figure 2B,D). The Chao-richness of the cecal microbiota of bacteriophage-treated birds (Group Cj_phg) was significantly different from mock-treated birds (Group Cj) by 5 dpt (*p* = 0.032; Figure 2B).

### Exposure to *Campylobacter*-Phage Selectively Reduces Proportions of *C. jejuni* Without Affecting the Wider Microbiota

Throughout the experiment the predominant bacterial phylum present in the ileal lumen was the Firmicutes with a median relative abundance (RA) of 83.091% (Supplementary Figure S5A). There were no differences in relative abundance between age-matched bacteriophage-treated (Group Cj_phg) and mock-treated birds (Group Cj) in this phylum (*p* ≥ 0.063, Wilcoxon test; Supplementary Figure S5A). The next most abundant phylum are the Proteobacteria where a difference at 4 dpt was observed (*p* = 0.016), but not on any other day (*p* ≥ 0.730, Wilcoxon test; Supplementary Figure S5A). Phyla-level composition of the cecal microbiotas of both groups were likewise dominated by Firmicutes (median 90.620% RA), and to a much lesser extent Proteobacteria (4.747% RA) (Supplementary Figure S5B). However, there was no difference in the relative abundance of Firmicutes or Proteobacteria between phage-treated (Group Cj_phg) and mock-treated birds (Group Cj) at any time post-treatment (*p* ≥ 0.178, *t*-test; and *p* ≥ 0.151, Wilcoxon test; respectively).

**FIGURE 3:**
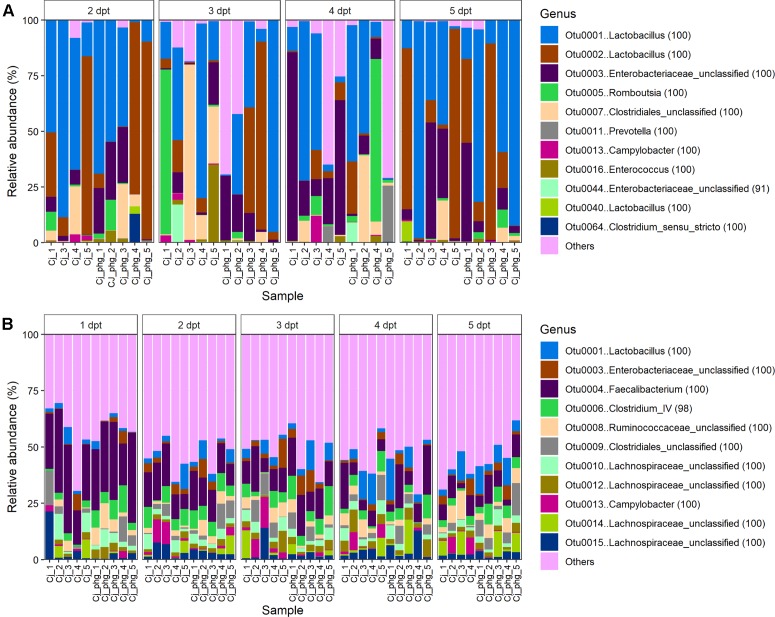
Bacteriophage treatment reproducibly only affects *C. jejuni* proportions. Stacked bar charts showing bacterial community compositions from the ileum **(A)** and ceca **(B)** of all birds. For clarity only the 11 most abundant OTUs are described with all other OTUs summarized as “other.”

At the OTU level, phage treatment did not affect the β-diversity (Bray Curtis distance) between communities of ileal lumen bacteria from age-matched phage-treated (Group Cj_phg) and mock-treated birds (Group Cj) at any time point (*p* = 0.106; AMOVA; Supplementary Figure S6A). The 11 most abundant OTUs in the ileum lumen are shown in Figure 3A. After the OTUs were filtered to include only those ≥1% of total reads, the only discriminative OTU between phage-treated and mock-treated birds identified using LEfSE (Segata et al., 2011) was OTU0013 [*Campylobacter* (100)] at 3 dpt (*p* = 0.009).

The 11 most abundant OTUs in the cecal lumen are shown in Figure 3B. For populations of cecal bacteria the β-diversity was not different at 1 to 4 dpt (*p* ≥ 0.088, Supplementary Figure S6B). However, bacterial populations could be distinguished at 5 dpt (*p* = 0.021). At 5 dpt OTU0009 [Clostridiales_unclassified (100)] was the sole discriminative OTU between the treatments (*p* = 0.027; LEfSE; Supplementary Figure S7D), which was present in significantly greater proportions in the mock-treated birds (Group Cj) relative to phage-treated birds (Group Cj_phg) (mean % RA = 1.211 and 6.668, respectively) (Figure 3A). Previous work from our laboratory identified an OTU with 100% DNA sequence identity to OTU0009 as being associated with *Campylobacter*-colonized birds [see OTU0073 in Connerton et al. (2018)]. Discriminative analysis of the cecal communities associated a greater abundance of *Camplylobacter* (OTU0013) with the mock-treated birds (Group Cj) at 3 and 4 dpi (*p* < 0.047; Supplementary Figures S7B,C, respectively). A positive association was observed between phage-treated (Cj_phg) birds at 1 dpt and OTUs 0006 and 0021, representing Clostridium IV (98) and Clostridiales_unclassified (100), respectively (*p* = 0.047; Supplementary Figure S7A and Figure 3B).

At 5 dpt the bacterial communities of the ceca or ileum of *Campylobacter*-free sentinel control birds could not be distinguished from mock-treated *Campylobacter*-colonized birds (Group Cj, *p* > 0.05; Supplementary Figures S6A,B), but cecal populations of bacteria in the phage-treated birds (Group Cj_phg) could be distinguished from non-colonized control birds (*p* = 0.014). Correspondingly, the OTUs that are associated with phage-treated treated birds (Group Cj_phg) are OTU0006 Clostridium IV (98) and OTU0009 Clostridiales_unclassified (100), which were also identified as discriminating phage-treated treated birds from mock-treated birds at 1 and 5 dpt, respectively (Supplementary Figure S8B). Although there was no difference in the β-diversity of non-colonized and mock-treated colonized birds in these cohorts, as expected OTU0013, representing *Campylobacter* (100), showed association with colonized birds, but conversely OTU0031, *Bifidobacterium* (100), showed association with the non-colonized control birds (Supplementary Figure S8A). This observation is also consistent with our previous study which demonstrates the differential association of an OTU with 100% DNA sequence identity with non-colonized birds compared to those 2 days post-colonization with *C. jejuni* at 20 days of age (NCBI database SRA study SRP133552; Connerton et al., 2018).

Interestingly, the low diversity of the ileal bacterial communities, relative to the cecal microbiota (see Figure 2A,B), is revealed in the observation that the top 11 most abundant OTUs across all ileal communities constitute 90.754% of reads, whereas the top 11 most abundant OTUs across all cecal communities account for a lower quotient of 48.670% reads (Figure 3A,B). We note that ileal samples showed some similarity to no template controls at phyla level (Supplementary Table 1), and that there was notable correspondence in the γ-proteobacteria [*Escherichia*/*Shigella* (100)] among the top 11 most abundant OTUs from ileal and cecal samples and the most abundant kitome/contaminant OTUs (Figure 3A,B; Supplementary Table 2).

## Discussion

The results described here provide further evidence of the efficacy of phage treatment to reduce the *Campylobacter* colonization of chickens. It has been widely suggested that an advantage of phage biocontrol over antibiotic use or other broad-spectrum types of therapy against pathogens that inhabit the intestinal tract, is the specificity of the bacteriophage selected for a particular host (Sulakvelidze and Barrow, 2004; Sulakvelidze and Kutter, 2004). This specificity is presumed to avoid the possibility of causing dysbiosis but recently this assumption has been challenged using a multi-phage treatment to affect collateral changes in the composition of the microbiota of rats with decreases in the abundance of *Blautia*, *Catenibacterium*, *Lactobacillus*, and *Faecalibacterium* species, and increases in the abundance of *Butyrivibrio*, *Oscillospira*, and *Ruminococcus* (Tetz et al., 2017). This is in contrast to directed studies using simulated gut microbial consortia (duodenum and ileum) containing a specific *Escherichia coli* as a bacteriophage target, where the impact of phage therapy was compared with ciprofloxacin treatment (Cieplak et al., 2018). Bacteriophage and antibiotic therapies were equally as effective in reducing the target *Escherichia coli* population by 2 to 3 log_10_ CFU ml^-1^ but notably the bacteriophage treatment had no measurable impact on non-target bacteria.

Although *Campylobacter* phages selected for the biocontrol of campylobacter in chickens appear to be confined to replication in *C. jejuni* and *C. coli* as hosts (Loc Carrillo et al., 2007), this discrimination had not previously been verified from the intestinal microbiota of *Campylobacter* colonized chickens. Frequency and abundance estimates of campylobacters and phages recovered from the ceca of commercial broiler chickens support the contention that production birds are often exposed to phage, and that phage presence coincides with a reduction in the mean *Campylobacter* cecal counts by approximately 1.8 log_10_ CFU g^-1^ (Atterbury et al., 2005). However, this study also demonstrated that phage which replicate on *Campylobacter* could be recovered in the absence of culture detectable *Campylobacter* host bacteria. The study left an open question as to whether the presence of phage under certain circumstances can drive *Campylobacter* populations below the culture detection limit (<2 log_10_ CFU g^-1^) or that phage infecting campylobacters could also replicate on alternative host bacteria present in the microbiota of chickens. Further motivation for the current study was to establish whether phage therapy under these circumstances constitutes a minimal targeted intervention that utilizes biocontrol agents that are not detrimental to the intestinal microbiota of farmed chickens, and to which consumers are already exposed.

Phage therapy of *C. jejuni* colonized chickens produced significant reductions in intestinal *C. jejuni* counts compared to mock-treated controls over 5 days. However, the introduction of the phages did not affect the structures of the cecal or ileal microbiotas of the birds based on calculations of α-diversity (inverse Simpson index). The richness (Chao) of the microbiota remained similarly indistinguishable until 4 dpt. At the phyla level no difference in the abundance of the major components were observed for the cecal microbiotas representing the greatest biomass, and at only one time point was any difference observed post-phage treatment for the ileal microbiotas (4 dpt). Analysis of differences in the relative abundance between phage-treated and mock-treated ileal and cecal community OTUs highlights significant differences in OTU0013 that represents the phage therapy target, *C. jejuni*. Additional to this, the cecal community member OTU0009 (Clostridiales_unclassified) showed significantly greater proportions in the mock-treated birds relative to phage-treated birds, as had previously been identified on the basis of the association of a DNA sequence identical OTU with *Campylobacter*-colonized birds (Connerton et al., 2018). The consistent association could be indicative of a key reliance for the corresponding clostridial organism(s) on high levels of *Campylobacter* colonization of the chicken gut. These observations further the idea that campylobacters can act as a hydrogen sink to improve the growth and competitive standing of specific clostridia (Kaakoush et al., 2015). The *Bifidobacterium* OTU0031 showed association with non-colonized sentinel birds, which is in contrast to 16S rRNA qPCR data reported by Thibodeau et al. (2015) that showed an increase in the molecular detection of *Bifidobacterium* sp. upon *C. jejuni* colonization. The authors noted that *Bifidobacterium* had previously been reported to hinder *C. jejuni* colonization (Ding et al., 2005; Santini et al., 2010), and that subtle effects may occur during *C. jejuni* colonization of chickens.

We observed the emergence of phage resistant *C. jejuni* post-phage treatment in this study as reported previously (Loc Carrillo et al., 2005; El-Shibiny et al., 2009; Fischer et al., 2013; Hammerl et al., 2014). We did not recover any phage resistant *C. jejuni* from the non-phage treated chickens despite the propensity of *C. jejuni* to undergo phase variation in genes leading to phage resistance (Aidley et al., 2017), and suggesting the observed phage escape mutation frequency of 10% in this experiment was a consequence of selection due to phage predation. The observed phage resistance frequency is within those reported previously of 1–14% (Loc Carrillo et al., 2005; El-Shibiny et al., 2009; Carvalho et al., 2010; Fischer et al., 2013; Hammerl et al., 2014). The phage treatment of chickens to reduce *Campylobacter* colonization has been demonstrated to be most effective over a 2–3 day period post-treatment (Loc Carrillo et al., 2005; El-Shibiny et al., 2009), since thereafter *Campylobacter* populations begin to recover. The continued impact of *Campylobacter* phage predation on the wider intestinal microbiota could not be assessed within the treatment timeframe examined in the current experiment (Connerton et al., 2018). However, when the time to slaughter after phage therapy was extended, the cecal *Campylobacter* levels were not reported to achieve the levels observed in non-treated controls (Fischer et al., 2013). This is likely due to reduced competitive fitness of the resistant types as described previously (Loc Carrillo et al., 2005; Scott et al., 2007a,b), and supports the supposition of Wagenaar et al. (2005) that the release of virulent *Campylobacter* phages into the environment would not constitute any greater risk.

Bacteriophage CP20 is a group II phage based on genome size and DNA sequence similarities. Group II phage are generally flagellotropic that require the host to be motile with a functional flagellar (Coward et al., 2006; Scott et al., 2007a,b; Baldvinsson et al., 2014; Lis and Connerton, 2016; Liang and Connerton, 2018). CP30A is a group III phage that in common with this phage group exhibits dependence on capsular polysaccharide structures (Sørensen et al., 2011; Lis and Connerton, 2016). The post-phage treatment *C. jejuni* isolates we identified as resistant to both phage classes will be of interest to examine with respect to their ability recolonize chickens and the nature of the mutation. Although we note they remain a minority population within the chicken gut even in the presence of bacteriophage controlling the wild type *C. jejuni* populations. These bacteria are likely at a competitive disadvantage to the wild type.

We have rigorously examined the microbiota of *Campylobacter* colonized chickens treated with either phages or a placebo to provide strong evidence for the lack of any collateral effect on the gut microbiome.

## Data Availability

The datasets generated for this study can be found in NCBI, NCBI Bioproject PRJNA506577 under the SRA study SRP170194.

## Author Contributions

IC designed the experiments. PR, PC, and IC executed the experiments, analyzed the data, and prepared the manuscript.

## Conflict of Interest Statement

The authors declare that the research was conducted in the absence of any commercial or financial relationships that could be construed as a potential conflict of interest.

## Footnotes

1 http://hmpdacc.org/resources/tools_protocols.php

2 https://www.mothur.org/wiki/MiSeq_SOP, (accessed 2018-10-05).

3 https://ftp.ncbi.nlm.nih.gov/blast/executables/LATEST/

## Supplementary Material

Figure S1 Rarefaction curves indicating coverage of *Campylobacter*-colonized (experimental) cecal bacterial communities. 16S rDNA bacterial communities from: **(A)** 1 day post-treatment (dpt); **(B)** 2 dpt; **(C)** 3 dpt; **(D)** 4 dpt; **(E)** 5 dpt. Cohort: **(i)** Group Cj **(ii)** Group Cj_phg.

Figure S2 Rarefaction curves indicating coverage of *Campylobacter*-free non-colonized (control) cecal bacterial communities. 16S rDNA bacterial communities from *Campylobacter*-free non-colonized control birds at 29 days-old, equivalent to 5 days post-treatment.

Figure S3 Rarefaction curves indicating coverage of ileal bacterial communities. 16S rDNA bacterial community from: **(A)** 2 day post-treatment (dpt); **(B)** 3 dpt; **(C)** 4 dpt; **(D)** 5 dpt. The non-phage treated cohort (Group Cj) are shown in panels Ai to Di, and the phage treated cohort (Group Cj_phg) in panels Aii to Dii.

Figure S4 *Campylobacter* phage titers in the ileum and colon post-treatment. Phage tiers were determined for CP20 **(A)** and CP30A phage **(B)** from ileal content and colon content **(C)**, CP20; **(D)**, CP30A). Filled squares indicate mean. Error bars indicate standard deviation.

Figure S5 Stacked barchart showing phyla-level microbiota composition for ileal and cecal communities. OTUs were filtered so that only Phyla present in more than one ileal or cecal community are included in the analysis. Figures in parenthesis in the key indicate bootstrap values for that taxonomic assignment generated in Mothur. All taxa shown in the key were detected in the ileum lumen including those of low abundance **(A)**. However, note that only Actinobacteria, Bacteria_unclassified, Bacteroidetes, Firmicutes, and Proteobacteria phyla were detected in cecal communities **(B)**.

Figure S6 Relationship between communties of gut bacteria from phage-treated and mock-treated birds. PCoA plot of Bray-Curtis distance between **A**) ileal (*R*^2^ = 0.41; **A,B**) cecal communities (*R*^2^ = 0.75; **B**).

Figure S7 Cecal bacterial taxa responsive to *Campylobacter*-phage treatment. OTUs were filtered to only include those representing at least 1% of the total reads before all communities were randomly subsampled an equal depth for unbiased comparison. Discriminatory OTUs were then described between age-matched groups using LEfSE. LDA refers to Linear Discriminant Analysis. 1 dpt **(A)**, 3 dpt **(B)**, 4 dpt **(C)**, 5 dpt **(D)**. There were no differential OTUs at 2 dp.

Figure S8 Gut bacterial taxa responsive to *Campylobacter*-colonization at 5 days post-treatment. OTUs were filtered as described for Supplementary Figure S7 and discriminatory OTUs identified using LEfSE. LDA refers to Linear Discriminant Analysis. Control-Group Cj comparison **(A)**, Control-Group Cj_phg comparison **(B)**.

Table S1 Description of phyla associated with no template controls. Due to potential for low biomass and dominant contaminant profile from ileum samples the kitome microbiota profile is included. Table text in parenthesis indicates Mothur taxonomy bootstrap values. Samples: kitome for all samples, excluding 1 dpt (kit Ai and kit Aii); kitome for 1 dpt (kit B); sequencing negative control for all samples, excluding 1 dpt (seq Ai and seq Aii); sequencing negative control for 1 dpt (seq B).

Table S2 Description of top 10 OTU associated with no template controls. Table text in parenthesis indicates Mothur taxonomy bootstrap values.
