# Supplementary figures and images for: Phage Biocontrol of Campylobacter jejuni in Chickens Does Not Produce Collateral Effects on the Gut Microbiota
Source: Front Microbiol. 2019 Mar 12;10:476. doi: 10.3389/fmicb.2019.00476 (PMC6423408; doi:10.3389/fmicb.2019.00476)

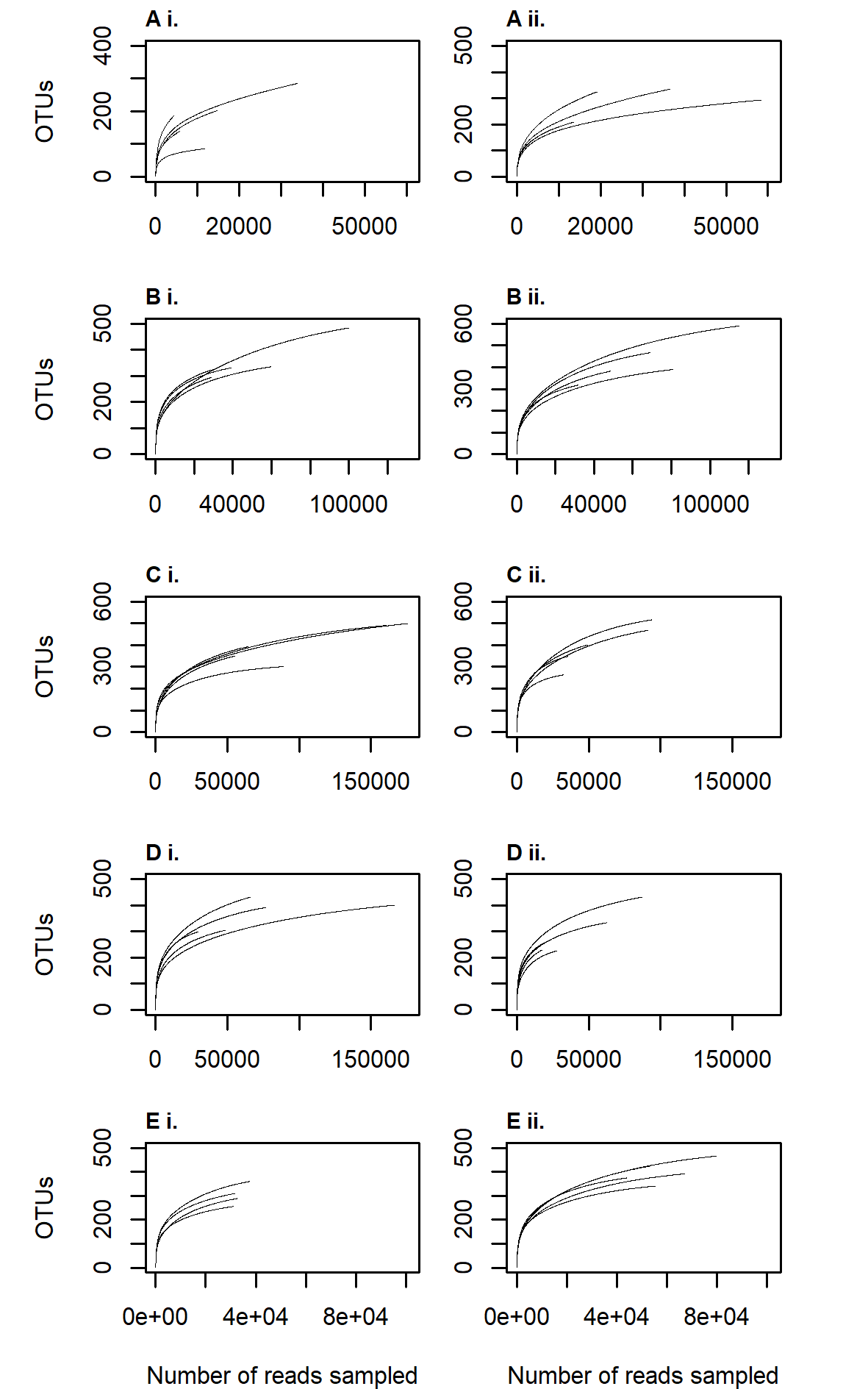

Supplement: Figure S1 — Rarefaction curves indicating coverage of Campylobacter-colonized (experimental) cecal bacterial communities. 16S rDNA bacterial communities from: (A) 1 day post-treatment (dpt); (B) 2 dpt; (C) 3 dpt; (D) 4 dpt; (E) 5 dpt. Cohort: (i) Group Cj (ii) Group Cj_phg. [file Image_1.TIFF]

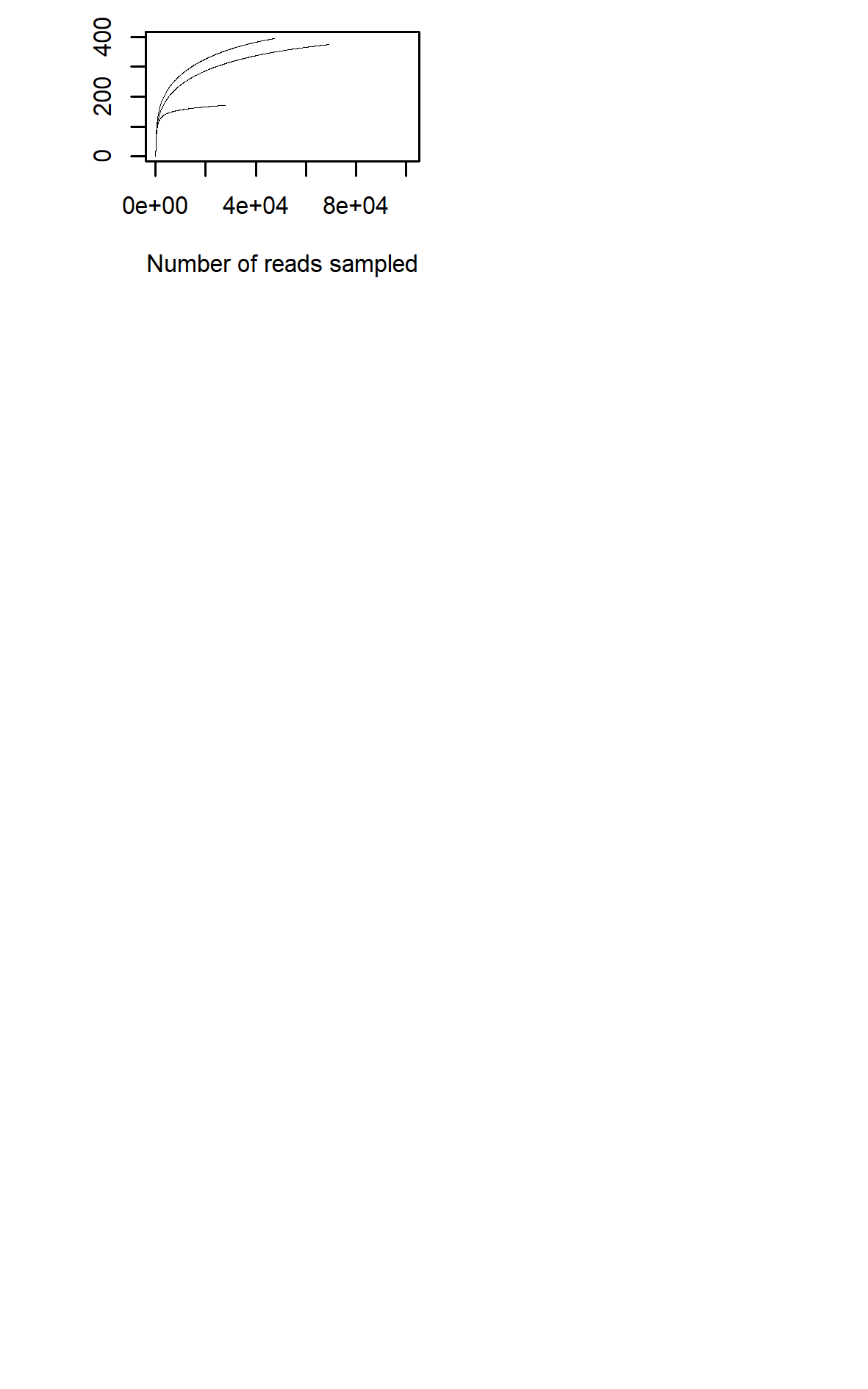

Supplement: Figure S2 — Rarefaction curves indicating coverage of Campylobacter-free non-colonized (control) cecal bacterial communities. 16S rDNA bacterial communities from Campylobacter-free non-colonized control birds at 29 days-old, equivalent to 5 days post-treatment. [file Image_2.TIFF]

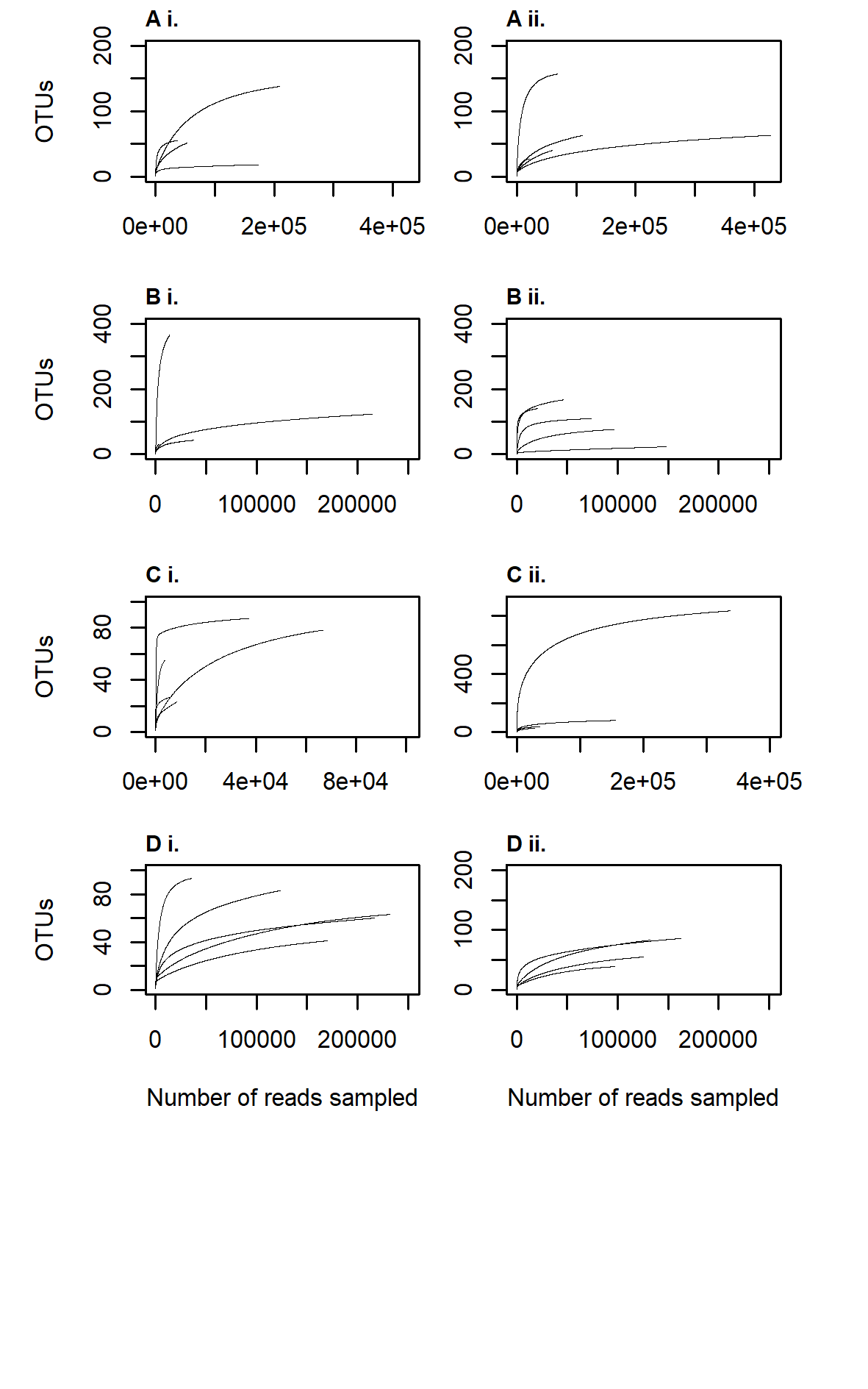

Supplement: Figure S3 — Rarefaction curves indicating coverage of ileal bacterial communities. 16S rDNA bacterial community from: (A) 2 day post-treatment (dpt); (B) 3 dpt; (C) 4 dpt; (D) 5 dpt. The non-phage treated cohort (Group Cj) are shown in panels Ai to Di, and the phage treated cohort (Group Cj_phg) in panels Aii to Dii. [file Image_3.TIFF]

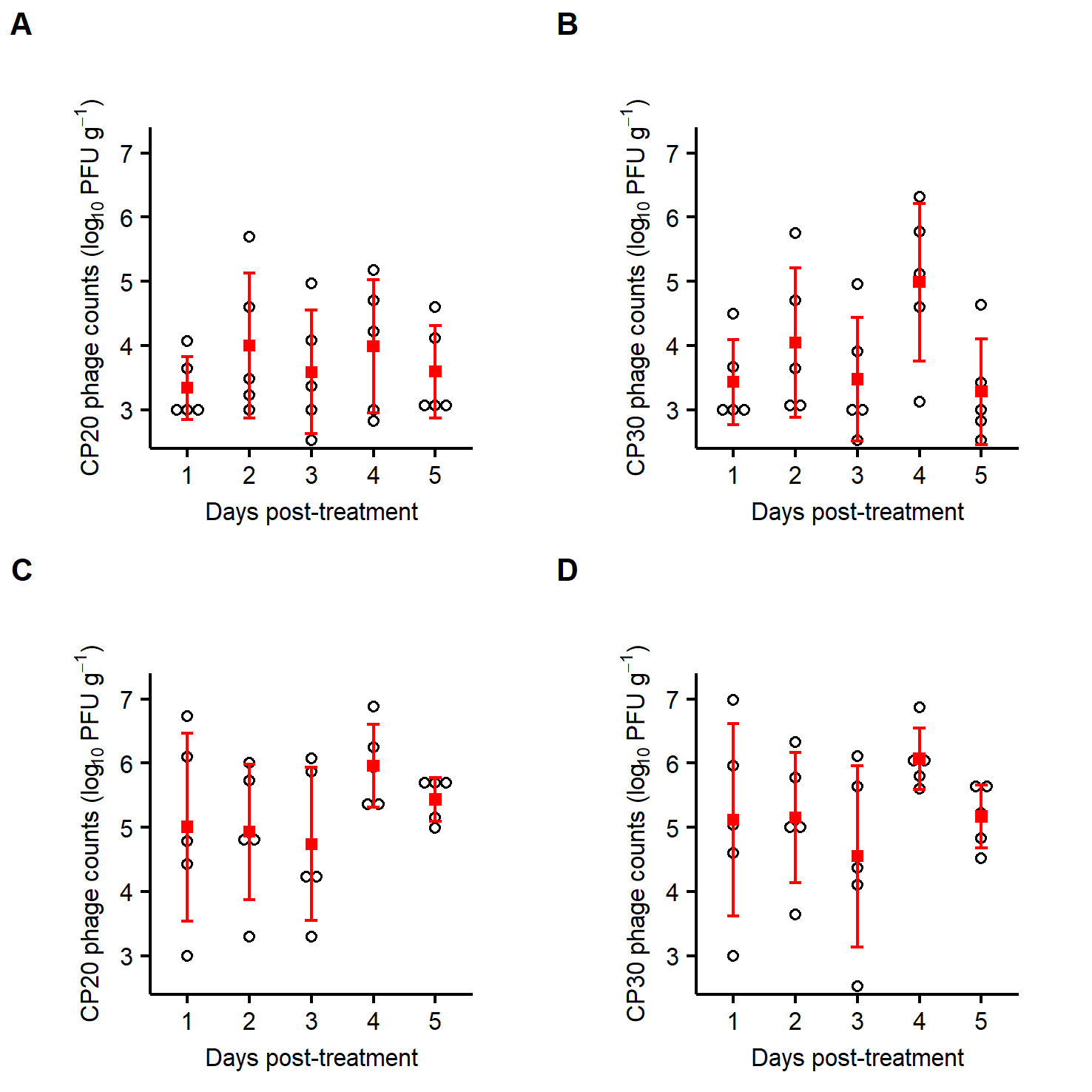

Supplement: Figure S4 — Campylobacter phage titers in the ileum and colon post-treatment. Phage tiers were determined for CP20 (A) and CP30A phage (B) from ileal content and colon content (C), CP20; (D), CP30A). Filled squares indicate mean. Error bars indicate standard deviation. [file Image_4.TIFF]

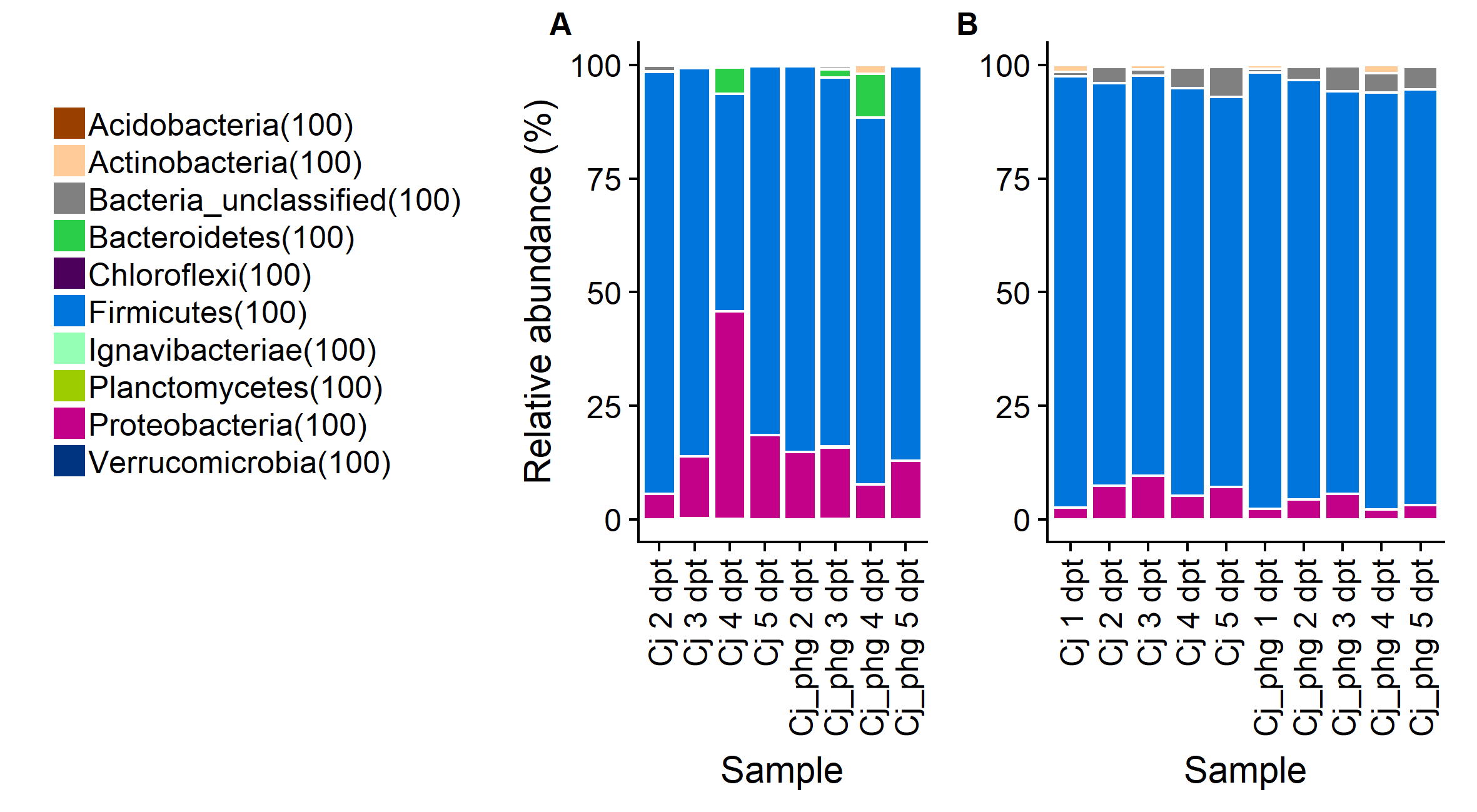

Supplement: Figure S5 — Stacked barchart showing phyla-level microbiota composition for ileal and cecal communities. OTUs were filtered so that only Phyla present in more than one ileal or cecal community are included in the analysis. Figures in parenthesis in the key indicate bootstrap values for that taxonomic assignment generated in Mothur. All taxa shown in the key were detected in the ileum lumen including those of low abundance (A). However, note that only Actinobacteria, Bacteria_unclassified, Bacteroidetes, Firmicutes, and Proteobacteria phyla were detected in cecal communities (B). [file Image_5.TIFF]

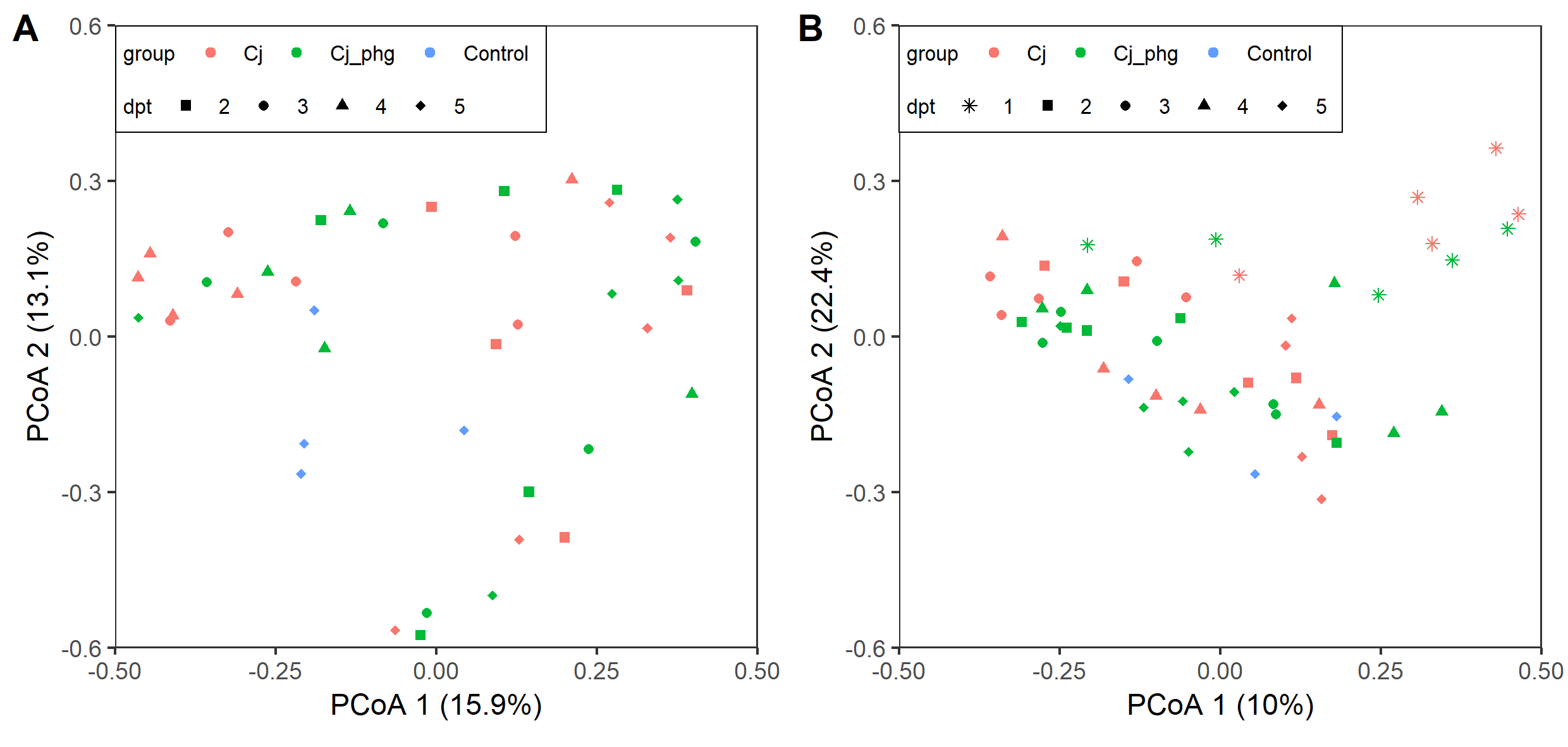

Supplement: Figure S6 — Relationship between communties of gut bacteria from phage-treated and mock-treated birds. PCoA plot of Bray-Curtis distance between A) ileal (R2 = 0.41; A,B) cecal communities (R2 = 0.75; B). [file Image_6.TIFF]

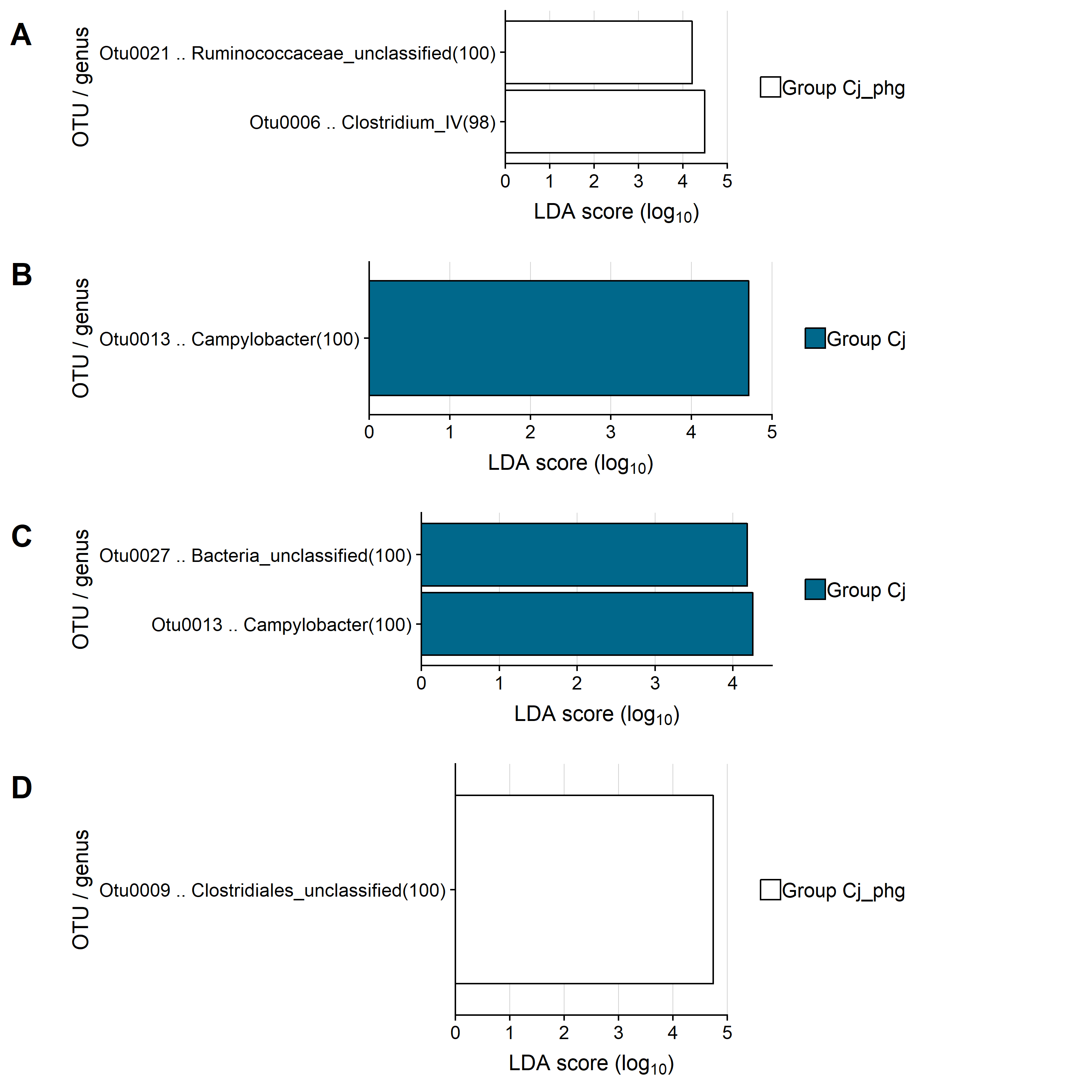

Supplement: Figure S7 — Cecal bacterial taxa responsive to Campylobacter-phage treatment. OTUs were filtered to only include those representing at least 1% of the total reads before all communities were randomly subsampled an equal depth for unbiased comparison. Discriminatory OTUs were then described between age-matched groups using LEfSE. LDA refers to Linear Discriminant Analysis. 1 dpt (A), 3 dpt (B), 4 dpt (C), 5 dpt (D). There were no differential OTUs at 2 dp. [file Image_7.TIFF]

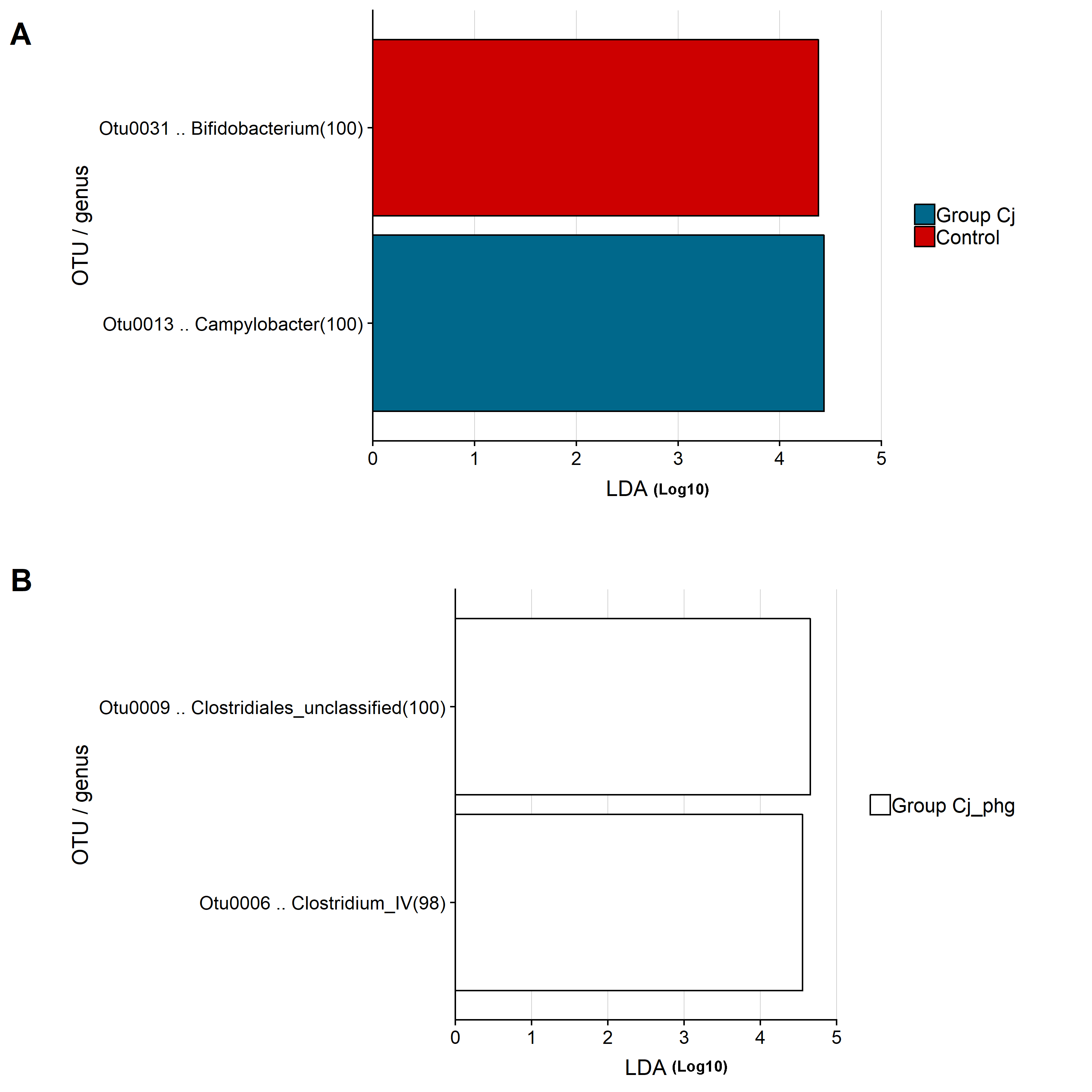

Supplement: Figure S8 — Gut bacterial taxa responsive to Campylobacter-colonization at 5 days post-treatment. OTUs were filtered as described for Supplementary Figure S7 and discriminatory OTUs identified using LEfSE. LDA refers to Linear Discriminant Analysis. Control-Group Cj comparison (A), Control-Group Cj_phg comparison (B). [file Image_8.TIF]
