# Supplementary material for: Phage Biocontrol of Campylobacter jejuni in Chickens Does Not Produce Collateral Effects on the Gut Microbiota
Source: Front Microbiol. 2019 Mar 12;10:476. doi: 10.3389/fmicb.2019.00476 (PMC6423408; doi:10.3389/fmicb.2019.00476)
Supplement: Table S1 — Description of phyla associated with no template controls. Due to potential for low biomass and dominant contaminant profile from ileum samples the kitome microbiota profile is included. Table text in parenthesis indicates Mothur taxonomy bootstrap values. Samples: kitome for all samples, excluding 1 dpt (kit Ai and kit Aii); kitome for 1 dpt (kit B); sequencing negative control for all samples, excluding 1 dpt (seq Ai and seq Aii); sequencing negative control for 1 dpt (seq B). [file Table_1.DOCX]

| **Phylum** | **kit Ai** | **kit Aii** | **kit B** | **seq Ai** | **seq Aii** | **seq B** |
| --- | --- | --- | --- | --- | --- | --- |
| Actinobacteria(100) | 0.3895451 | 2.1676301 | 0.00000 | 2.7673681 | 1.369863 | 0.000000 |
| Bacteria_unclassified(100) | 0.3895451 | 0.3853565 | 0.00000 | 0.3603344 | 0.000000 | 7.088608 |
| Bacteroidetes(100) | 0.6534305 | 5.9730250 | 0.00000 | 11.8694148 | 2.739726 | 18.227848 |
| Cloacimonetes(100) | 0.0000000 | 0.0000000 | 0.00000 | 0.0000000 | 0.000000 | 1.518987 |
| Firmicutes(100) | 6.4526263 | 34.6339114 | 23.63636 | 21.4759297 | 86.301370 | 4.810127 |
| Planctomycetes(100) | 0.0000000 | 0.0000000 | 0.00000 | 0.0000000 | 0.000000 | 8.101266 |
| Proteobacteria(100) | 92.1148530 | 56.8400771 | 76.36364 | 63.5269530 | 9.589041 | 53.924051 |
| Thermotogae(100) | 0.0000000 | 0.0000000 | 0.00000 | 0.0000000 | 0.000000 | 3.797468 |
| Verrucomicrobia(100) | 0.0000000 | 0.0000000 | 0.00000 | 0.0000000 | 0.000000 | 2.531646 |
