# Supplementary material for: Phage Biocontrol of Campylobacter jejuni in Chickens Does Not Produce Collateral Effects on the Gut Microbiota
Source: Front Microbiol. 2019 Mar 12;10:476. doi: 10.3389/fmicb.2019.00476 (PMC6423408; doi:10.3389/fmicb.2019.00476)
Supplement: Table S2 — Description of top 10 OTU associated with no template controls. Table text in parenthesis indicates Mothur taxonomy bootstrap values. [file Table_2.DOCX]

| **OTU** | **Phylum** | **Class** | **Order** | **Family** | **Genus** | **Kit Ai** | **kit Aii** | **kit B** | **seq Ai** | **seq Aii** | **seq B** |
| --- | --- | --- | --- | --- | --- | --- | --- | --- | --- | --- | --- |
| Otu0001 | Firmicutes(100) | Bacilli(100) | Lactobacillales(100) | Lactobacillaceae(100) | Lactobacillus(100) | 0.307866298 | 2.36030829 | 0.4545455 | 0.021620063 | 0.000000 | 0.00000 |
| Otu0002 | Firmicutes(100) | Bacilli(100) | Lactobacillales(100) | Lactobacillaceae(100) | Lactobacillus(100) | 0.069112842 | 3.37186898 | 0.0000000 | 1.340443932 | 0.000000 | 0.00000 |
| Otu0003 | Proteobacteria(100) | Gammaproteobacteria(100) | Enterobacteriales(100) | Enterobacteriaceae(100) | Enterobacteriaceae_unclassified(100) | 91.122141242 | 15.51059730 | 76.3636364 | 56.839146728 | 9.589041 | 53.92405 |
| Otu0004 | Firmicutes(100) | Clostridia(100) | Clostridiales(100) | Ruminococcaceae(100) | Faecalibacterium(100) | 1.897461674 | 11.41618497 | 10.9090909 | 0.007206688 | 0.000000 | 0.00000 |
| Otu0006 | Firmicutes(100) | Clostridia(100) | Clostridiales(100) | Ruminococcaceae(100) | Clostridium_IV(98) | 0.006282986 | 1.83044316 | 1.3636364 | 0.014413376 | 0.000000 | 0.00000 |
| Otu0007 | Firmicutes(100) | Clostridia(100) | Clostridiales(100) | Clostridiales_unclassified(100) | Clostridiales_unclassified(100) | 0.012565971 | 0.04816956 | 0.0000000 | 0.000000000 | 0.000000 | 0.00000 |
| Otu0008 | Firmicutes(100) | Clostridia(100) | Clostridiales(100) | Ruminococcaceae(100) | Ruminococcaceae_unclassified(100) | 0.000000000 | 0.86705202 | 0.4545455 | 0.007206688 | 0.000000 | 0.00000 |
| Otu0009 | Firmicutes(100) | Clostridia(100) | Clostridiales(100) | Clostridiales_unclassified(100) | Clostridiales_unclassified(100) | 0.483789897 | 0.04816956 | 0.0000000 | 0.000000000 | 0.000000 | 0.00000 |
| Otu0010 | Firmicutes(100) | Clostridia(100) | Clostridiales(100) | Lachnospiraceae(100) | Lachnospiraceae_unclassified(100) | 0.012565971 | 0.52986513 | 0.0000000 | 0.007206688 | 0.000000 | 0.00000 |
| Otu0011 | Bacteroidetes(100) | Bacteroidia(100) | Bacteroidales(100) | Prevotellaceae(100) | Prevotella(100) | 0.006282986 | 0.00000000 | 0.0000000 | 4.151052176 | 2.739726 | 0.00000 |
| Otu0012 | Firmicutes(100) | Clostridia(100) | Clostridiales(100) | Lachnospiraceae(100) | Lachnospiraceae_unclassified(100) | 0.961296808 | 0.43352601 | 0.0000000 | 0.000000000 | 0.000000 | 0.00000 |
